# Understanding Collective Discontents: A Psychological Approach to Measuring Zeitgeist

**DOI:** 10.1371/journal.pone.0130100

**Published:** 2015-06-26

**Authors:** Anne Marthe van der Bles, Tom Postmes, Rob R. Meijer

**Affiliations:** 1 Department of Social Psychology, University of Groningen, Groningen, the Netherlands; 2 Department of Psychometrics and Statistics, University of Groningen, Groningen, the Netherlands; Cajal Institute, Consejo Superior de Investigaciones Científicas, SPAIN

## Abstract

Over the last decade, several countries around the world developed a collective sense of doom and gloom: Their Zeitgeist could be characterized as one of decline. Paradoxically, in some countries, such as the Netherlands, this collective discontent with society seems to exist despite high levels of individual well-being. Current psychological research informs us about why individuals would feel unduly optimistic, but does not account for a collective sense of decline. The present research develops a novel operationalization of Zeitgeist, referred to as a general factor *Z*. We conceptualize Zeitgeist as a collective global-level evaluation of the state (and future) of society. Three studies confirm that perceptions of the same societal problems at the personal and collective level differed strongly. Across these studies we found support for a hypothesized latent factor *Z*, underlying collective-level perceptions of society. This *Z*-factor predicted people’s interpretation of new information about society that was presented through news stories. These results provide a first step in operationalizing and (ultimately) understanding the concept of Zeitgeist: collectively shared ideas about society. Implications for policy are discussed.

## Introduction

Notwithstanding high levels of individual well-being, in several Western countries the general outlook on the state of society is decidedly pessimistic. People indicate that they are troubled by several discontents with the collective welfare in society: there are nation-wide problems with poor security, a lack of social cohesion, bad economic prospects, and so forth. However, judgments made at this collective level can be markedly different and independent from personal level judgments: the majority of individuals who believe that *we* are unhappy can, at the same time, claim that *I* am happy. Thus, the aggregate of personal perceptions may bear little resemblance to collective perceptions. The present research seeks to develop a better operationalization of such collective societal discontents.

We hypothesize that judgments about *specific* societal issues (e.g., safety, education, immigration) are guided by a more *global* affective evaluation of the state of society. These global affective evaluations are abstract, collective level judgments (i.e., a sense that “we” are doing well or are doing badly). These global affective evaluations, we suggest, are strongly guided by the perceived social consensus. In this way, a climate of (dis)content can emerge, an aspect of what is sometimes referred to in popular terms as Zeitgeist. The purpose of the present research was to attempt to develop an operationalization of this Zeitgeist. The utility of such an instrument is that, in future, it would allow us to gain a better understanding of collective societal discontents. The paper proposes and tests two related measures of Zeitgeist, operationalized as a latent factor *Z*. The research shows that *Z* can predict several outcomes, including what news stories people are inclined to believe and what inferences they draw from particular incidents about society and people in general.

### Societal Discontents

For the past decade or more, many Western societies have experienced a sense of doom and gloom. The situation in the Netherlands provides an extreme example: approximately since 2001, public intellectuals, commentators and politicians have presented the country as “rotten to the core”: In public debate and the media, the image portrayed has been one of a deeply divided country suffering endemic problems with the welfare state, state services, and democracy itself [1,2]. Indeed, approximately three-quarters of Dutch citizens appear to be convinced that “society” is moving in the wrong direction [1,2]. The same phenomenon, although less extreme, can be witnessed in many other Western societies. In 2013, 65% of US citizens and 68% of UK citizens were dissatisfied with the way things are going in their country; figures that have been relatively stable since 2007 [3]. In Australia, pessimism about society is a more recent phenomenon: since 2010 trust in the federal government fell sharply and a sense of pessimism about the future has increased [4]. Apparently, across various countries a *consensus* has emerged that society is doing badly.

This phenomenon is particularly puzzling when contrasted to statistics that suggest that private well-being is high. In case of the Netherlands, 82% of Dutch people state that they are happy with their personal life [1]. And 67% of US and 65% of UK citizens describe their personal economic situation as good [3]. In Australia between 2010 and 2014, 87–89% of people indicated being happy in their personal life [4]. Thus, while a large majority of individuals is of the opinion that society is in decline, at the same time there is a large majority of individuals who report to be happy with their personal life. How can such deeply felt societal discontents be reconciled with a happy citizenship and (relatively) good living-conditions, within the same societies and (to some extent) within the same individuals?

In understanding this phenomenon, it is important to note that the sense of gloom focuses on the abstract collective level (society, the country, the people, “us”) whereas the sense of well-being focuses on more concrete personal outcomes (my personal circumstances, my life, “I”). These different levels of abstraction, we suggest, are linked to distinct social realities. Through (inter)national surveys, we can construct relatively well how people view their personal lives, based on the information about themselves which they are willing to share with interviewers or survey agencies. International organizations such as the Organisation for Economic Co-operation and Development (OECD) provide information on various indicators of personal-level well-being, which is then aggregated to infer OECD countries’ (economic) state. But we argue that the collective level of well-being is only partially captured by aggregating personal-level statistics: personal well-being can be quite independent of discontents with society as a whole. This is because *collective*-level discontents express dissatisfaction about *us*, about our society: Collective discontents (with education, immigration, individualism, the welfare state, etc.) are about the perceived collective problems that the group as a whole is supposed to suffer from. These discontents may be completely independent of people’s personal problems or the well-being of individuals.

### Judgments about others and judgments about society

How can we start to conceptualize collective societal discontents? One literature that is important here focuses on why people on average tend to be more positive about themselves in comparison to others. Such social comparative judgments are studied extensively in social psychology, as for example the Better-than-Average and comparative optimism effects (see e.g. [5–8]). This research has specifically focused on explaining why people on average perceive themselves to be better than others on a range of personal characteristics (e.g. better drivers [9] or more polite than others [10]) and less at risk of a negative life event happening to them [11]. This discrepancy between judgments about the self and others is explained by motivated as well as non-motivated processes (for reviews see e.g. [5,7]). Motivated explanations focus on desires for self-enhancement: people have unreasonably rosy views of their personal life [6]. Non-motivated explanations have mainly focused on cognitive biases caused by the fact that people often have more information about themselves than they do about others or see the self as more central in the jugdment process [7].

The most common method used to investigate the effects in this literature is through direct comparison. Participants are asked to judge: how sociable am I, compared to the average student [7]. This method has been developed because the indirect method, asking seperately how sociable I am and how sociable I think the average student is, has several disadvantages. The direct method is important because it allows researchers to optimally answer the dominant question in this field: why are personal judgments relatively rosy? Could this be because of motivational factors (self-enhancement), because of cognitive biases, or both?

As a result of methodological choices as well as of these guiding questions, the field has focused less on the question how judgments about others are made. The underlying assumption tends to be that we have less knowledge about others than about ourselves [12]. But whilst it is true that people tend to know less about *concrete* others than they do about themselves, it is also true that in many cases people believe they can form very good judgments of others in a more global sense. The prime example is when people judge members of an outgroup: here, socially shared stereotypes are likely to inform judgments in a systematic fashion [13]. In this sense, judgments about a *collective* object or generalized other can differ starkly from judgments of concrete others. Therefore we suggest that judgments at this collective level are qualitatively different from personal-level judgments about the self or about concrete others. A discrepancy between these levels of judgments is thus intriguing but not necessarily informative for understanding why or how people judge collective-level objects: both levels of judgments are influenced by distinct types of information. So to understand the pervasive gloominess about the state of society apparent in various countries, we propose it is important to study people’s *collective* judgments about issues or characteristics of their society, independently of the parallel personal judgments they might make.

### Prior research into collective-level judgments

If we want to study collective judgments or perceptions it is important to know how to operationalize them. From the literature, we were unable to distill a consensus about the best operationalization of collective-level judgments or perceptions. In much research, operationalizations of collective-level judgments simply aggregate personal-level judgments in a variety of different ways. For example, sociological research on the transmission of values between parents and their children has attempted to operationalize Zeitgeist [14,15]. In this research Zeitgeist was operationalized as the most frequently existing (i.e. modal) individual value in the population. Similarly, in communication research investigating public opinion climate from the perspective of the Spiral of Silence theory [16], public opinion polls of people’s personal judgments are described as the straightforward way to make inferences about the public opinion climate [17]. In both cases a general collective concept (Zeitgeist or public opinion climate) is inferred from personal-level judgments.

But as reviewed above, the psychological literature already shows that personal and aggregate-level judgments tend to be very different. Accordingly, we argue that it would be erroneous to make inferences about, for example, the (perceived) safety of society from aggregate personal judgments of safety. The perception of society as unsafe is likely to be determined by very different factors than personal safety perceptions. Indeed, research on risk perceptions has shown that societal-level and personal-level risk judgments are distinct types of judgments with different psychological antecedents (e.g., [18,19]). For example in research on climate change risk perceptions, Van der Linden [19] showed that knowledge about the causes and consequences of climate change and response-behavior reducing climate change uniquely predicted societal-level but not personal-level risk perceptions. In contrast, personal experience with extreme weather events predicted personal-level, but not societal-level risk perceptions.

Recent studies of cross-national and cross-cultural comparisons have also distinguished more systematically between levels of judgment of the kind which we explore in the current research. This research shows that perceived collective-level characteristics in a nation or culture can be independent from personal-level prevalence of these characteristics or values. Research on personality and stereotypes of national character, for example, showed that collective-level perceptions of national personality characteristics are largely unrelated to aggregated personal characteristics [20,21]. More interestingly, personality and national stereotypes *both* predict behaviors, albeit different kinds [22].

Similar conclusions can be drawn from the cross-cultural literature. Chiu and colleagues [23] have attempted to measure collective-level perceptions of cultural values, which they refer to as intersubjective judgments. Intersubjective perceptions are conceptualized as shared perceptions: respondents are asked to what extent a representative member of the group would endorse certain values (intersubjective judgment) and this is contrasted to respondents’ personal endorsement. Studies of intersubjective values found that they are quite distinct from individual-level values (e.g., [24–27]). For example, American participants perceived other Americans to be more individualistic (vs. collectivistic) and Polish participants perceived other Poles to be more collectivistic (vs. individualistic), while actual (personal) levels of endorsement of these values did not differ between American and Polish participants [27].

In sum, both cross-national and cross-cultural research confirms the possibility of meaningfully operationalizing judgments at the collective level. The present research applies these insights not to national stereotypes or cultural values, but rather to judgments about society as a whole. Specifically, we will distinguish personal-level and collective-level judgments about important public issues that could affect respondents’ life directly, but that could also characterize the state of society as a whole.

### Operationalizing Zeitgeist

The common perceptions of the problems that exist in many Western countries are not just very negative, they also seem to generalize across a very broad range of outcomes: the sense of decline extends to society as a whole, not just one concrete part of it. How can one account for the relatively abrupt emergence of such a broad spectrum of societal discontents within a broad segment of society? Societal discontents (as well as societal satisfactions) appear to flourish in a particular societal climate or *Zeitgeist*: a pervasive, consensual perception of society. Society as a whole appears to be rotten, and this taints every concrete judgment about society. How can this be operationalized from a social psychological perspective?

In the present research, we propose that specific societal discontents (e.g., with education, crime, etc.) stem from a global feeling of discontent with society—something which one could term negative Zeitgeist. Our reasoning is that this negative Zeitgeist functions as some form of collective prejudice against one’s own society: a shared preconceived opinion that things in society are bad (or good, as the case may be). We propose that this Zeitgeist colors *particular* societal (dis)contents; since these (collective) judgments are not necessarily based on concrete facts or actual personal experiences. In other words, when the Zeitgeist concerning the state of society is very negative, this taints judgments of particular societal issues ranging from, say, crime levels to satisfaction with service levels. Thus, Zeitgeist should affect collective perceptions of any component of society. Abstract societal topics should be especially strongly affected by the Zeitgeist, since for these topics (for example “individualism”) other more concrete sources of information on society’s state are less readily available [28].

**Fig 1:**
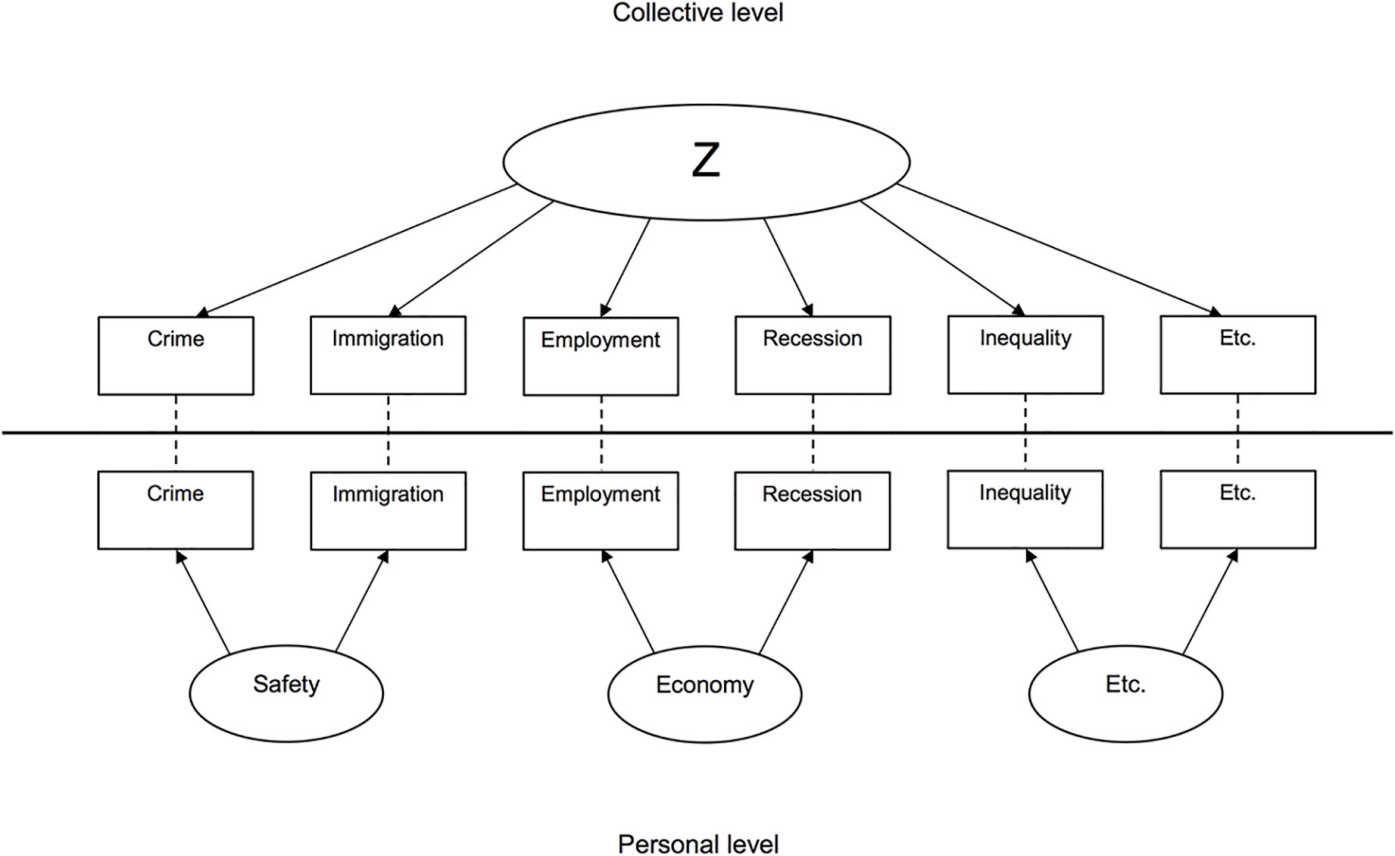
Conceptual model of Zeitgeist.

Putting both these elements together, one could operationalize Zeitgeist as a general factor (or “Z”) that predicts a range of distinct collective judgments (cf. the top half of Fig 1). Statistically, this approach can be compared with the g-factor in intelligence research where G is an underlying “general intelligence” factor that predicts performance on particular IQ tests [29]. In our approach, Z is an underlying “general (dis)content” factor that predicts particular societal discontents and satisfactions.

Furthermore, since we are interested in the influence of Zeitgeist on perceptions of society as a whole, one needs to ask individuals about their perceptions of collective societal discontents and satisfactions. Following the literature on social comparative judgments reviewed above, we argue that the state of society can be inferred if one asks questions about the conditions faced by the average or modal citizen (i.e. the “generalized” other). Such perceptions of the challenges faced by us, collectively (e.g., the average person in my society feels unsafe), can be markedly different from one’s personal-level experiences (e.g., I feel unsafe).

In the present research, we therefore contrast collective judgments with personal judgments of societal issues (e.g., “crime is a problem in the life of the average citizen” vs. “crime is a problem in my life”). Since personal judgments are influenced by concrete personal experiences and (direct) social comparisons in the many different areas of everyday life, we expect that personal-level judgments of various societal issues would have strong interrelations when issues concern the same area of life, but lower interrelations when issues concern different areas of life (and therefore, for a range of topics as a whole, see Fig 1’s bottom half). This contrasts with collective societal judgments, which we assume to be more strongly influenced by the Zeitgeist.

### Overview of the Present Research

In this paper, we develop and test a new method to measure Zeitgeist regarding the state of society. Specifically, we designed two complementary measures of personal and collective perceptions of the same societal issues. These two measures were tested in three studies, across two different societal contexts: the Netherlands (Study 1 and 2) and the United States (Study 3).

The first measure was created to assess the perceived prevalence of relatively concrete personal and collective experiences on a scale that would allow us to make direct comparisons between personal and collective perceptions: Participants were asked to estimate how many out of the last 30 days they (personal level) or the average person in their country (collective level) encountered specific societal problems (e.g. crime, the recession). We reasoned that this method of measuring the prevalence of problems would echo the personal level questions asked in (inter)national surveys (e.g., Eurobarometer, World Value Survey) to gather information on the prevalence of societal problems. In addition, it would offer a framework within which one could make “direct” comparisons between the personal and collective perceptions: While my personal problems with crime in my neighborhood are relatively rare, most other people have (or the average person has) these problems more frequently. This measure is used in Study 1 and 3.

A limitation of this type of measure, however, is that it is less suitable to measure abstract concerns that people might have (e.g., with individualism). We therefore developed a measure using a more conventional format in social psychological research: statements on a 7-point Likert scale. These evaluative statements concerned abstract concepts and issues that are important for a well-functioning society (e.g. corruption, inequality). They resembled statements about the state of society which are frequently encountered in daily life, as voiced by politicians or through the media: for example, “Corruption is a problem in our society”. These collective-level statements were contrasted with personal-level statements about the same issues, but more concretely formulated (e.g., Many people I know act corruptly). This measure is tested in Study 2 and 3.

We used these measures to address three central questions. First, we investigate aggregated personal- and collective-level perceptions (Study 1–3). Based on the presumed prevalence of a negative Zeitgeist in both the Netherlands and the US, we expect these perceptions to differ to the extent that people will be more negative about collective life than about their personal life. Finding these differences would not only highlight the presence of this negative Zeitgeist, but also show that aggregated personal and collective perceptions of the same social issues are qualitatively different.

Second, we explore the factor structures of personal and collective perceptions (Study 1–3). We expect to find evidence for a latent general factor *Z* that underlies collective-level perceptions of society, in line with our conceptualization of Zeitgeist. For personal-level perceptions we predict that these will show less general coherence and form clusters according to various areas of personal life (e.g., safety, economy). But we have no specific predictions for the number of factors at this personal level (not least because our measures constitute a broad range of issues: we did not anticipate any specific factor structure a priori). By contrast, at the collective level we expect to find one single factor or dimension that explains substantial variance in particular judgments. This would be consistent with the idea that there is a coherent Zeitgeist at the heart of negativity about issues as diverse as crime, immigration, individualism, lack of respect, etc.

Third, we investigate the predictive validity of *Z* (Study 2 and 3). We expect that collective self-prejudices would influence how people interpret and attribute new information they receive about their society: Any “news” about the state of society should, to some extent, be colored by such prejudices. This is examined by having participants indicate whether they think several pessimistic news headlines could be true (Study 2 and 3), and to examine how responsibility for negative events is attributed to society (Study 2).

## Study 1

### Method

#### Participants and procedure

Fifty male and 126 female Dutch university students (*M*_age_ = 21.13; age range: 17–50 years) were approached in canteens or after participation in another study to fill in a short paper-and-pencil questionnaire. During informed consent they were informed that the research explored experiences of people who live in the Netherlands.

#### Ethics statement

This research was approved by the Ethical Committee Psychology of the University of Groningen. Study 1, written informed consent was obtained. In accordance with local ethical standards, people aged 16 and over are considered capable to decide whether or not to participate in research, so local ethical guidelines do not require consent by parents or guardians. In Study 2 and 3, which were conducted as online surveys, written informed consent was obtained (and included a statement which stated that by starting the questionnaire respondents agreed to participate, as is in line with local standards).

#### Prevalence estimates

Participants estimated the prevalence of societal problems in the past 30 days on two different dimensions: for themselves (personal) vs. for the average Dutch person (collective). The 12 problems were: Criminality; Alcohol abuse; Immigrants; Loitering teens; The recession; Personal enrichment or fraud; The government; The police; Foreigners; Money shortage; Offensive behavior by people they know; and Offensive behavior by strangers. We selected these specific problems because they reflected societal problems that at the time were discussed in politics and media in the Netherlands.

First, we instructed participants to think about their personal lives: “think for example about the conversations you have, the things you do, and the people and situations you encounter in your daily life”. Subsequently, they estimated the number of days they encountered the 12 problems (ranging from 0 to 30) for the past 30 days in their personal lives (personal dimension; α = .74). Next, participants were instructed to think about the average Dutch person: “What does the life of a Dutch person look like during 30 days? Think about the conversations Dutch people have, the things they do, and the people and situations they encounter.” Following this introduction, participants estimated the number of days that the average Dutch person encountered the 12 problems during the last 30 days (collective dimension; α = .92). This prevalence estimates measure is reported in full in S1 Appendix. In addition, age and sex were assessed. Finally, participants were debriefed and thanked for participating in this research.

#### Additional methodological detail

We originally attempted to manipulate participants’ perceptions of the possible (future) state of Dutch society (positive vs. negative vs. both positive and negative). On a separate page before the prevalence estimates measures, participants read a short text that framed the state of the country as either an upward trend (positive), a downward trend (negative), or not a clear trend (both positive and negative) since the 1980s, e.g. “The following questions concern the situation in the Netherlands. We are interested in this topic because Dutch people have lived in adversity for the past few years [vs. prosperity for the past few years vs. have lived in prosperity for too long].” However the manipulation check was non-significant and no systematic effects of the manipulation on the DV’s approached significance; we concluded that the manipulations were ineffective and these will not be discussed further. Furthermore, in addition to judgments about the *past* 30 days, we also asked participants to estimate the prevalence of societal problems during a period of 30 days some years in the future (in the month of their graduation). These future judgments were largely the same as those for the present. Because they add so little insight into the process, future judgments are ignored in this paper.

Before the prevalence estimates measure, we exploratively included potential predictors of Z: belief in a just treatment (14 items, adapted from [30]), satisfaction with life (5 items, adapted from [31]), and optimism (10 items, adapted from [32]). Because the emphasis in this paper is on the factor structure, these variables are not taken into account here. Correlations between these variables and collective- and personal-level prevalence estimates are presented in Table A in S1 Supplementary Materials, for interested readers.

### Results

#### Analytic strategy

Upon data screening, two participants were excluded from analyses for not completing the questionnaire. Furthermore, multivariate outlier analysis with Mahalanobis distance (see e.g. [33]) identified eight multivariate outliers (participants with inconsistent patterns of scores on items constituting key variables, e.g., scoring 7 on both normal and reversed-scored items) which were excluded from further analyses. All subsequent analyses were based on N = 166 participants. First, mean differences were explored. Second, exploratory factor analyses (EFAs) examined how many factors could be extracted on the basis of conventional criteria in EFA (i.e., parallel analysis [34] in R 3.0.2 with the nFactors package and (theoretical) coherence of the extracted factors). We chose EFA rather than confirmatory factor analysis (CFA) because we did not have a priori predictions for a specific factor structure of personal-level prevalence estimates.

#### Inspection of differences in means

We examined differences in mean prevalence estimates between personal and collective judgments using repeated measures analyses of variance [ANOVA]. This ANOVA showed a strong significant main effect of the personal-collective dimension, *F*(1,165) = 261.47, *p* < .001, η_p_^2^ = .62. While participants on average estimated to have encountered the societal problems in their life on 1.98 days (95% CI [1.62, 2.33]), they estimated the average Dutch person to have encountered these problems on 6.45 days (95% CI [5.78, 7.12]). Thus as expected, people were more negative about collective life than about their personal lives.

#### Exploratory factor analyses [EFAs]

We conducted EFAs to explore whether estimates of societal problems would cluster into one (collective level) or multiple (personal level) factors. Principal Axis Factoring with oblique Promax rotation (assuming that factors could be correlated, [35]) was performed with IBM SPSS Statistics 20 on the prevalence estimates per dimension (personal vs. collective). Initial screening indicated that the items referring to problems with the recession and money shortage were very highly correlated, pointing to redundancy issues. We therefore chose to exclude the item “Money shortage” from the EFAs, thereby enhancing interpretation of the factor solutions. Thus, two EFAs were performed on 11 problem-items per dimension. For the purpose of the research question and limitations of space, it is sufficient to summarize the key findings.

For *personal estimates*, the best fitting factor solution extracted four factors (see Table 1; Note that parallel analysis was not entirely conclusive on whether the best fitting factor structure had four or three factors. We choose to report the four-factor solution because of relatively better interpretability of the extracted factors). Eigenvalues of these factors were 3.33, 1.51, 1.35, and 1.15 and the total explained variance was 66.79%. Factor loadings ranged between .41 and .97. However, several communality coefficients (i.e., the percentage of variance in a given variable explained by all the factors together) were below .40 and therefore considered poor (see e.g., [36]). In sum, the personal-level four-factor solutions had a reasonably good fit to the data.

**Table 1:** EFA Pattern and Structure Coefficients of Prevalence Estimates (Promax; Study 1).

		Personal				Collective	
Variable	Factor 1	Factor 2	Factor 3	Factor 4	*h* ^*2*^	Factor 1	*h* ^*2*^
Foreigners	**.72** (.70)		(.25)	(.28)	.49	**.80**	.65
Immigrants	**.70** (.57)				.37	**.84**	.70
Loitering Teens	**.53** (.64)	(.31)	(.46)	(.27)	.45	**.79**	.63
The police	**.41** (.56)	(.35)	(.34)	(.40)	.38	**.78**	.61
The recession		**.75** (.67)			.49	**.64**	.41
The government	(.39)	**.71** (.74)			.56	**.68**	.47
Fraud		**.56** (.58)	(.37)		.40	**.59**	.35
Criminality	(.44)	(.29)	**.97** (.98)		.96	**.77**	.59
Alcohol abuse			**.48** (.44)		.24	**.66**	.43
Indecency–known	(.31)		(.31)	**.97** (.95)	.92	**.72**	.52
Indecency–strangers	(.41)	(.30)		**.49** (.56)	.39	**.69**	.48
Eigenvalue	3.33	1.51	1.35	1.15		6.27	
Percentage of variance	30.31	13.69	12.30	10.48		56.97	
Correlations: Factor 1	–	.41	.42	.37			
Correlations: Factor 2		–	.29	.27			
Correlations: Factor 3			–	.26			

*Note*. Structure coefficients are in parentheses. Coefficients in bold load on factor (> .32, see [33]). Coefficients smaller than .25 are not displayed. *h*2 = communality coefficient.

In contrast, for *collective judgments* a single factor was extracted. This single factor had an eigenvalue of 6.27 and explained 56.97% of the variance (see Table 1). The factor loadings were good to excellent, ranging between .59 and .84. Most communality coefficients were sufficiently high as well; only the item “fraud” had a communality value below .40. Thus, for the collective-level dimension, the single-factor solution seemed to be a good fit to the data. In line with our expectations, the EFAs suggested that multiple factors underlie estimates of the prevalence of societal problems at the personal level, while one factor underlies estimates of the same societal problems at the collective level (for the average Dutch person).

#### Variance explained by Z

In order to examine the hypothesis that collective-level judgments would be accounted for by a single underlying dimension of *Z*, we examined the proportion of variance in the items explained by a single factor solution at both personal and collective level. For personal-level judgments, results showed that on average 23% of the variance in the items was accounted for by single-factor *Z*. For collective-level items, on average 54% of the variance was accounted for by *Z*. Hence, collective-level estimates bear evidence of a strong latent factor *Z*, whereas at the personal level, there is only weak evidence of such a latent factor.

### Discussion

The results of Study 1 showed that as predicted, participants estimated the prevalence of societal problems in the life of the average Dutch person to be higher than in their personal lives. Moreover, in contrast to personal-level estimates, collective-level estimates clustered together in one factor in EFA. This single latent factor in turn accounted for a higher amount of variance in the variables. Together, these results are consistent with our prediction that Zeitgeist as represented by a latent factor *Z* underlies specific collective-level judgments about society.

A measure based on prevalence estimates of societal problems has specific advantages: its unit of measurement (days) has the same meaning across dimensions, which enhances comparability across measures (see e.g. [13]). Additionally, this measure focuses on relatively concrete problems that people encounter both in their daily life and in the abstract (e.g., crime as a concrete experience vs. as a social construct). However, a limitation of this methodology is that it ignores a second class of societal discontents: abstract notions that cannot be expressed in absolute numbers, such as “trust in government”. In Study 2, we therefore tested an alternative method to measure this second aspect of *Z*, which captures these abstract societal discontents.

## Study 2

In Study 2 we investigated abstract-level judgments about ideas, values and institutions in society. Such discontents may crop up in public debate as blanket assertions about society: “we” have lost trust in the government, society has problems with immigrants or solidarity among citizens is declining. Again, we suggest that at the collective level, these abstract judgments are influenced by one underlying evaluation of the state of society: a Zeitgeist. In contrast, personal and more concrete judgments should be predicted more strongly by personal experiences, which may cluster together in various factors reflecting various domains of life. In order to assess these predictions, we developed a measure of collective perceptions of society that asks participants to judge the same issues on different dimensions: that is, on a personal and collective level, and with these same issues framed in more abstract and more concrete terms. For theoretical reasons, we are most interested in examining the personal-level concrete judgments and collective-level abstract judgments. Exploratively, we also included personal-abstract and collective-concrete judgments. These additional analyses (which also examined the differences in means between all dimensions and the influence of abstract vs. concrete wording in the items) are presented in S1 Supplementary Materials.

Furthermore, Study 2 explores the predictive validity of *Z*. We expect that Zeitgeist would affect people’s spontaneous interpretation of new events and new information about their society: any new incidents and stories would have to be interpreted in such a way that they become aligned with the current Zeitgeist. Thus, we predict that *Z* will influence this impromptu interpretation. If the Zeitgeist is negative, pessimistic news headlines would be more likely to be perceived as true than optimistic ones. Furthermore, when confronted with individual examples of negative behavior, *Z* should predict whether these negative events are attributed to general characteristics of society and human nature.

### Method

#### Participants and design

Participants were 255 members of a representative panel of the Dutch population. They completed an online questionnaire distributed by a commercial agency in return for a monetary reward (the equivalent of approx. $2,-). The sample consisted of 146 men and 109 women, who ranged in age from 18 to 80 years old (*M* = 49.5, *Med* = 54).

#### Procedure and materials

Participants received a link to an online survey about life in the Netherlands. The introduction page of the questionnaire provided participants with information about the research purposes and an informed consent agreement. Before the main measures, several constructs were included for explorative reasons and to aid assessment of convergent and discriminant validity in future studies. These constructs were (all measured on 7-point scales): Optimism (10 items; adapted from [32]); general social trust (3 items, adapted from [37]); *social trust in other groups* (4 items, adapted from [38]); political trust (4 items; adapted from [37]); neighbourhood safety (4 items, adapted from [37]); identification with the Netherlands (4 items; [39]); and state of the country (2 items: “In general, would you expect life for most people in the Netherlands to become better or worse?” and “To what extent is life for most people in the Netherlands better or worse than in the year 2000?”). Correlations between these variables and collective- and personal-level evaluative statements are presented in Table B in S1 Supplementary Materials, for interested readers. The main measures were presented to participants in the same order as described below. After completion, participants were given a brief explanation of the purpose of the study and were thanked for their cooperation.

#### Personal and collective perceptions of (dis)content

We measured participants’ judgments of 14 societal topics on a personal (concrete) and a collective (abstract) judgment dimension. The societal topics were selected to reflect a broad range of issues considered important in contemporary (Dutch) society, both for the functioning of society in general and as perceived challenges to society today. The topic selection of topics generally important for society was inspired by Haidt and colleagues’ moral foundations theory, which reflects domains that are important in collective (moral) life: Harm/Care, Fairness/Reciprocity, Ingroup/Loyalty, Authority/Respect, and Purity/Sanctity [40,41]. A further set of issues was derived from current public debates on the state of Dutch society (e.g., trust in government, immigration). We aimed to balance the valence of the evaluative statements, by including positively (+) as well as negatively framed topics. Thus, the 14 societal topics included in our measure were: Violence, Care (+), Honesty (+), Corruption, Injustice by others, Injustice by governmental agencies, Inequality, Trust (+), Social Cohesion (+), Egotism, Immigration, Loyalty (+), Lack of respect, and Lack of decency. Each topic was assessed with two items. For example, the resulting items for the societal topic “egotism” were (translated into English): “In my personal life, my experience is that people act mostly out of self-interest” (personal-concrete) and “Egotism is a problem in society” (collective-abstract). The personal-concrete wording reflects concrete experiences with selfishness that people might encounter in their day to day life. The collective-abstract wording is of the form in which it enters the public debate.

All evaluative statements were measured on a scale from 1 *strongly disagree* to 7 *strongly agree*, with 4 *neither agree*, *nor disagree*. Personal and collective items were measured in two separate blocks. Within these blocks, all items were randomized. First, participants received instructions to think about themselves and their personal lives: “think for example about the conversations you have, the things you do, and the people and situations you encounter in your daily life”. Participants subsequently indicated agreement with items at the personal level. Next, participants received instructions to think about Dutch society and indicate agreement with collective-level items: “think for example about topics Dutch people tend to talk about, the things that Dutch people do, and the situations and individuals that Dutch people encounter in society”. All items are reported in full in S1 Appendix.

#### Predictive validity: newspaper headlines and news reports

In order to explore whether Z predicted participants’ impromptu interpretation of new information about society, participants were first asked to indicate the extent to which they thought 14 *newspaper headlines* could be true. These headlines consisted of seven pairs, providing the opposite claim on the same topic: for example, “Criminality in the Netherlands strongly increased in 2011” (a negative headline) and “Criminality in the Netherlands strongly decreased in 2011” (positive). Two subscales were computed: *negative headlines* (α = .71) and *positive headlines* (α = .76). The headlines were rated on a scale from 1 *very untrue* to 7 *very true*, with 4 *neutral*.

Participants then read three short *news reports*, “such as one could encounter daily in the newspapers, on the internet, or on television”. These were inspired by news stories which had made headlines in the regional or national media a few years ago, about a man who laid dead in his house for two years before he was found by the police, about a man who had beaten a dog to death, and about ambulance personnel who were attacked by bystanders. After reading each report, participants indicated to what extent the cause of this event lay with (i) those involved in the situation, (ii) current society, and (iii) humanity, on a scale from -3 *not at all true* to 3 *very true*, with 0 *neutral*. Three subscales were computed across the three news reports (three items each): *situational attribution* (low reliability, α = .27), *societal attribution* (α = .56), and *attribution to humankind* (α = .72). Finally, demographic variables were assessed: age, sex, nationality, education level, and employment status.

### Results

#### Analytic strategy

During preliminary screening for outliers seven participants were excluded. Five participants were excluded because they took less than 5 minutes to complete the study, which on average took participants approximately 19 minutes (5% trimmed mean). One participant was excluded for completing only the first part of the questionnaire (i.e. not the main variables). Furthermore, one outlier was detected after screening for multivariate outliers by computing Mahalanobis distance [33]. All further analyses were thus conducted on responses of *N* = 248 participants. First, EFAs examined the factor structure underlying personal(-concrete) and collective(-abstract) perceptions of society. Given that we did not have predictions for the specific factor structure of personal-level judgments, we again chose to examine factor structure using EFA. Second, regression analyses examined whether interpretation of news headlines and incidents could be predicted by *Z*. Analyses examining mean differences between personal and collective judgments are reported in S1 Supplementary Materials. The mean differences were in line with the results of Study 1 and showed that participants were more negative (and less positive) about collective life compared to their personal lives.

#### Exploratory Factor Analyses (EFA)

As in Study 1, Principal Axis Factoring with Promax rotation was used. For *personal-concrete judgments*, three factors were extracted with eigenvalues of 5.56, 1.66, and 1.13 (see Table 2). The total amount of variance explained by this factor structure was 59.63%. Communality coefficients tended to be quite low, with several values below .40. The variables seemed not well-defined by this factor solution, with two variables not loading on any factor (loadings < .32; other factor loadings ranged from .35 to .99). Thus, we concluded that for personal-concrete judgments, the factor solution was suboptimal.

**Table 2:** EFA Pattern and Structure Coefficients of Personal-Concrete and Collective-Abstract Judgments (Promax; Study 2).

	Personal-Concrete				Collective-Abstract		
Variable	Factor 1	Factor 2	Factor 3	*h* ^*2*^	Factor 1	Factor 2	*h* ^*2*^
Loyalty	**.88** (.81)	(.41)	(.31)	.67	(.43)	**.78** (.78)	.61
Care	**.75** (.70)	(.32)	(.32)	.49	(.41)	**.72** (.73)	.53
Trust	**.74** (.76)	(.46)	(.37)	.58	(.48)	**.76** (.79)	.63
Honesty	**.73** (.75)	(.44)	(.38)	.56	(.45)	**.80** (.81)	.65
Social cohesion	**.71** (.64)	(.26)	(.30)	.42	(.33)	**.77** (.72)	.53
Lack of respect	**.35** (.57)	(.50)	.29 (.54)	.43	**.71** (.72)	(.41)	.52
Injustice by others	.29 (.54)	(.53)	(.51)	.40	**.62** (.70)	(.48)	.50
Inequality	(.34)	**.99** (.84)	(.38)	.74	**.64** (.63)	(.32)	.39
Corruption	(.30)	**.61** (.61)	(.40)	.39	**.60** (.64)	(.40)	.41
Egoism	.29 (.49)	**.43** (.55)	(.30)	.36	**.77** (.70)	(.29)	.50
Injustice by government	.30 (.50)	**.35** (.53)	(.37)	.34	**.60** (.63)	(.38)	.40
Violence	(.29)	(.41)	**.99** (.88)	.78	**.65** (.60)	(.27)	.37
Immigration	(.44)	(.44)	**.67** (.72)	.53	**.42** (.44)	(.26)	.19
Lack of decency	(.42)	.31 (.54)	.31 (.53)	.37	**.71** (.73)	(.42)	.53
Eigenvalue	5.56	1.66	1.13		5.77	1.95	
Percentage of variance	39.73	11.85	8.06		41.24	13.95	
Correlations: Factor 1	–	-.56	-.49		–	-.54	
Correlations: Factor 2		–	.56				

*Note*. Structure coefficients are in parentheses. Coefficients in bold load on factor (> .32, see [33]). Coefficients smaller than .25 are not displayed. *h*2 = communality coefficient.

In contrast, for *collective-abstract judgments*, two factors were extracted with eigenvalues of 5.77 and 1.95, explaining 55.19% of the variance in the data (see Table 2). Item-valence seemed to have influenced the factor structure to create a “method factor”: negative variables loaded on one factor, positive variables on another. The factors were highly correlated with each other at-.54. Factor loadings were all excellent, ranging between .60 and .80, except for the item Immigration (.42). This item stands out due to its relatively low communality coefficient (.19), suggesting that it might not be a close fit to the factor. In all, we concluded that this two-factor solution has good fit for collective-abstract judgments.

The fact that negatively and positively framed variables formed a method factor can be due to characteristics (i.e., valence) on item-level, but could also indicate that judgments about positive characteristics of society are to some extent independent of judgments about negative characteristics. For now, we will take this valence distinction into account in our further analyses, and assess the amount of variance in the items that is accounted for by this two-factor representation of *Z*.

#### Variance explained by Z

Again, we investigated the influence of *Z* on the judgments of specific issues by calculating the amount of variance in the items that was accounted for by *Z* (i.e., in this model *Z-negative* and *Z-positive*). Results show that on average, 42% of the variance in negative collective judgments was accounted for by *Z*, compared to 36% for negative personal judgments. For positive collective judgments, on average 59% of the variance was accounted for by *Z*, compared to 54% for positive personal judgments. Thus, in line with Study 1 these results suggest the strongest influence of a latent factor *Z* on collective(-abstract) judgments.

#### Exploring predictive validity

Finally, we examined whether participants’ interpretation of news headlines and news reports could be predicted by Zeitgeist, as represented by their scores on the latent factor *Z*. For this we focused only on the negative items, because we were specifically interested in the relation between *discontent* and negative news and events in this Dutch context. Following the factor structure extracted from EFA, aggregated scores were computed for two negative personal judgment factors (Factor 1: Injustice by others, Inequality, Corruption, Egoism, Injustice by Government (α = .72) and Factor 2: Violence, Immigration (α = .77)) and one collective judgment factor (Factor 1: Injustice by others, Inequality, Lack of respect, Corruption, Egoism, Injustice by government, Violence, Immigration, and Lack of decency (α = .86)). Regression analyses were used to determine the influence of personal and collective judgments on the interpretation of news headlines and attributions. We expected the collective level to be the best predictor in line with hypotheses concerning *Z*. Table 3 presents the results.

**Table 3:** Regression Analysis for Predictors of Negative and Positive Headlines and Attribution of Causality to Society and Humanity in Reports (Study 2).

	Negative headlines		Positive headlines		Reports–Society		Reports–Humanity	
	B (SE)	β	B (SE)	β	B (SE)	β	B (SE)	β
Constant	2.47 (.20)***		4.78 (.25) ***		2.68 (.37)**		1.86 (.47) ***	
Personal 1	-.02 (.04)	-.03	.05 (.05)	.08	-.07 (.07)	-.07	.04 (.09)	.03
Personal 2	.02 (.03)	.05	.08 (.04)	.14*	.00 (.05)	.00	.09 (.07)	.10
Collective	.55 (.05)	.64***	-.50 (.06)	-.54***	.58 (.09)	.44***	.49 (.12)	.31***
*R* ^2^	.41***		.23***		.17***		.14***	

*Note*. Personal 1 = mean personal-concrete judgments of Injustice by others, Inequality, Corruption, Egoism, Injustice by Government; Personal 2 = mean personal-concrete judgments of Violence, Immigration; Collective = mean collective-abstract judgments of Injustice by others, Inequality, Lack of respect, Corruption, Egoism, Injustice by government, Violence, Immigration, Lack of decency.

* *p* ≤ .05

** *p* < .01

*** *p* < .001.

We first examined the influence of the judgment dimensions on *negative news headlines*. Only collective judgments were a significant predictor, β = .64, *t*(244) = 11.10, *p* < .001, while personal judgments did not predict participants’ interpretation of negative news headlines. So, people with higher collective-level reports of societal problems were more likely to interpret negative news headlines as truthful, while personal-level perceptions of problems were unrelated. Collective judgments explained 41% of the variance in scores on negative news headlines (*F*(3, 244) = 56.17, *p* < .001). Similar results were found for *positive news headlines*, although there was less variance explained (*R* ^*2*^ = .23, *F*(3, 244) = 24.53, *p* < .001). Again, collective judgments significantly (and negatively) predicted participants’ interpretation of positive news headlines, β = -.54, *t*(244) = -8.21, *p* < .001. The second personal-level judgment factor was a significant (positive) predictor as well, β = .14, *t*(244) = 2.28, *p* = .02, but had a quite small unique contribution to the explained variance compared to collective-level judgments (*sr* ^*2*^ = .02 vs. *sr* ^*2*^ = .21 respectively).

The influence of *Z* on the *attribution of causality in news reports* was examined using the same approach (see Table 3). The items for attribution of causality to the persons involved in the news reports combined to an unreliable scale (α = .27), and will therefore be left out of subsequent analyses. With respect to *societal attributions*, only collective judgments were a significant predictor, β = .44, *t*(244) = 6.41, *p* < .001, (*R* ^*2*^ = .17, *F*(3, 244) = 16.12, *p* < .001). Thus, higher reports of societal problems predicted people’s attribution of causality in these sensational news reports to society. The results for *attributions to humankind* were again broadly similar (see Table 3). Only collective judgments significantly predicted attributions to humankind (β = .31, *t*(231) = 4.27, *p* < .001 and *R* ^*2*^ = .14, *F*(3, 231) = 11.98, *p* < .001). In sum, these results suggest that collective(-abstract) judgments representing *Z* are the best predictors of interpretations and attributions of new information about society.

### Discussion

The results of Study 2 replicated the patterns we found in Study 1. These results provide further evidence for a general factor *Z*, reflecting Zeitgeist in collective-abstract judgments about society. EFA showed that for collective-abstract judgments, a well-fitting two-factor structure could be defined; this reflected not only our hypothesized general factor *Z*, but also a “method factor” constructed through the influence of item valence (positive vs. negative). In contrast, EFA did not reveal a well-fitting factor solution for personal-concrete judgments. Furthermore, a two-factor representation of *Z* (a positive and a negative factor) accounted for most variance in collective-abstract items, compared to personal-concrete items. Further evidence for the conceptualization of Zeitgeist is provided through the exploration of its predictive validity. We found that participants’ judgments of collective-abstract issues, representing *Z*, were the strongest or only predictor of their impromptu interpretation and attribution of new information about society. While the measures we used in Study 1 and 2 have important differences, both seem to be able to reflect the gloom about society that is felt in the Netherlands. In order to gain understanding of the relation between these measures, we directly compared them by including both in Study 3.

## Study 3

Study 3 has two main aims: first, it explores the relation between the prevalence estimates of concrete societal problems and the (more conventional) judgments of societal issues applied to (concrete) personal life versus (abstract) collective life. By combining both measures in one study, we aim to learn more about how these judgments of societal issues are connected to each other and we can directly compare their utility.

Second, Study 3 transfers these methods of measuring perceptions of society from the context of Dutch society to American society. By changing the societal context of our research, we hope to explore the generalizability of our conceptualization of Zeitgeist and our newly designed methods for measuring it. Since in the US the general perception of the state of society also seemed to be gloomy (e.g. [3]), we believed this to be an interesting comparison country for the results we obtained in the Netherlands.

### Method

#### Participants and design

Participants were 287 (167 female, 120 male) members of the American general public, who ranged in age from 18 to 82 years old (*M* = 34.7, *Med* = 31, *SD* = 12.5). The sample was predominantly White (81.9%), with a minority of African Americans (5.6%), Hispanics (5.9%), and Asians (4.9%). The data was collected shortly before the US 2012 presidential election; 56.1% (161) of participants intended to vote for Obama/Biden (Democratic Party), 27.2% (78) for Romney/Ryan (Republican Party), and 16.7% (48) participants either intended to vote for a third party candidate, were undecided, or indicated not to care about the elections.

#### Procedure and materials

Participants were invited through Amazon MTurk to complete an online questionnaire. They received $0.50 for participation. The study started with information about the research purposes and an informed consent agreement. The questionnaire generally replicated the design of Study 2; the constructs were measured in order as described below. As in Study 2, we included the variables that may help to inform us about convergent and divergent validity in later research: optimism [32], neighbourhood safety (4 items, adapted from [37]), general social trust [37], social trust in other groups [38], and political trust [37]. Correlations between these variables and collective- and personal-level judgments are presented in Table C in S1 Supplementary Materials, for interested readers. After completion, participants were given a brief description of the study and thanked.

#### Collective perceptions of society

Collective perceptions of society were assessed by combining the methods of Study 1 and 2: prevalence estimates of specific societal problems and (dis)agreement with evaluative statements about general societal topics. Both measures are reported in full in S1 Appendix.

*Prevalence estimates*: Prevalence of 18 societal problems was judged in one’s own life (personal level) and for the average American (collective level), using the method described in Study 1. The problems that were included were partly based on Study 1 but adjusted to include societal issues that were discussed in the period prior to the US presidential election: Crime, Personal safety, Loitering teens, Immigration, Health care provision, Global warming, The economy, The recession, Money shortage, Unemployment, The government, The police, Corruption or fraud, Discrimination, Obesity, Alcohol or drugs abuse, Offensive behavior by strangers, and Offensive behavior by friends/acquaintances.

*Evaluative statements*: Participants were asked to judge statements about 12 general societal topics. Based on Study 2, we included the following topics: Violence, Care (+), Honesty (+), Corruption, Injustice, Inequality, Trust (+), Social cohesion (+), Egoism, Loyalty (+), Lack of respect, and Lack of decency. Each topic was measured on a personal-concrete and a collective-abstract judgment-dimension. We also exploratively included personal-abstract and collective-concrete judgments. These results are generally in line with those of Study 2 and not reported here. The topics were measured on a scale from 1 *strongly disagree* to 7 *strongly agree*, with 4 *neither agree*, *nor disagree*.

Items at the personal and collective level were measured in two separate blocks, which were counterbalanced. At the personal level, participants first received instructions to think about their personal day-to-day experiences: “Think for example about the conversations that you have, the things that you do, and the people and situations you encounter in your daily life”. Participants then were asked to indicate their (dis)agreement with the personal-concrete evaluative statements. After this, participants completed the prevalence estimates at personal level.

At the collective level, participants were instructed to think about experiences in present-day society: “Think about the issues that other Americans talk about, the things that other Americans do and the people and situations that other Americans encounter in society”. Subsequently, participants indicated the extent to which they agreed with the collective-abstract evaluative statements, and after this, they completed the prevalence estimates at collective level.

#### Predictive validity: newspaper headlines

We measured participants’ interpretation of newspaper headlines to explore the predictive validity of *Z*. This measure was based on the measure we used in Study 2, but adapted to the American context and altered in the sense that we used 10 positive and negative single statements (as opposed to paired statements in Study 2). Participants indicated the extent to which they thought these headlines could be true on a scale from 1 = *completely untrue* to 7 = *completely true*, with 4 = *neither true*, *nor untrue*. Examples of the headlines were: “U.S. crime rates increase in 2012” and “Increasing number of Americans in debt”. The full scale (six items reflecting negative developments in society and four items reflecting positive developments in society) had low reliability and inspection of means, standard deviations, and correlations suggested that several items were understood differently then we intended. We therefore decided to leave aside one negative item (designed to resemble a form of symbolic threat, “Fewer Americans celebrate Thanksgiving”) and the four positive items (e.g., “Trust in government is growing”) and instead use five negative items to form a *negative headlines* scale (α = .61). Finally, demographic variables were assessed: age, sex, ethnicity, level of education, voting intentions, and political views.

### Results

On the basis of preliminary screening for outliers with Mahalanobis distance (see e.g. [33]) 12 participants with incoherent response patterns on key variables (e.g., consistently answering 7 on both normal and reversed items) were excluded. All further analyses were conducted on *N* = 275 participants. The analytic strategy followed that of Studies 1 and 2. However, for analyzing the results of Study 3 we used CFA to test the one factor *Z* model and the amount of variance explained by *Z* in collective-level (vs. personal-level) judgments.

#### Inspection of differences in means

We conducted one-way repeated measures ANOVA’s separately for the prevalence estimates and the negatively- and positively-framed evaluative statements. Results of Study 1 and 2 were globally replicated. For the *prevalence estimates*, participants estimated to have personally encountered societal problems on 5.28 [4.72, 5.83] out of the last 30 days (a score much higher than that in Study 1, also on individual items concerning crime, a.o.). However, the average American was estimated to have encountered the same problems about twice as much: 10.45 [9.67, 11.24] out of the last 30 days, *F*(1,273) = 228.33, *p* < .001, η_p_^2^ = .46.

For *negatively-framed evaluative statements*, participants disagreed that negative topics affected their personal life, (*M* = 3.20 [3.08; 3.32]), but on average agreed that these topics affected collective life, (*M* = 4.49 [4.37, 4.60]), *F*(1,274) = 384.15, *p* < .001, η_p_^2^ = .58. For *positively-framed statements*, participants agreed that these topics affected their personal life (*M* = 5.12 [5.00, 5.24]) more than they affected the life of Americans in general (*M* = 4.20 [4.09, 4.31]), *F*(1,274) = 263.86, *p* < .001, η_p_^2^ = .49. Thus as expected, participants were more negative and less positive about societal issues in collective life than in their personal lives, both when responding to statements and when estimating concrete numbers.

#### Relations between dimensions

The correlations between factors in all studies (1–3) are reported in full in Table D in S1 Supplementary Materials. Here we focus only on the correlation between the different Z estimates based on prevalence estimates and evaluative statements. This correlation was moderate to strong (*r* = .41), suggesting that there was (limited) overlap. The difference between these two estimates could either be due to them tapping into different kinds of judgments or information about what society is, but it is also likely that the different measurement scales reduced the overlap.

#### Confirmatory factor analyses (CFA): assessing variance explained by *Z*

We used a structural equation modelling technique (CFA) to test the *Z*-model and the amount of variance explained by *Z* in the prevalence estimates and collective(-abstract) and personal(-concrete) statement-judgments. The variance *R* ^*2*^ accounted for by *Z* indicates the extent to which a general factor influences perceptions of particular societal problems, and is our main outcome variable. We predicted that this *R* ^*2*^ would be higher for collective- than for personal-level judgments. Estimates of model fit also indicate to what extent a single dimension could be identified. Thus, we inspected the standardized root-mean-square residual (SRMR; value < .08 indicates reasonable fit; [42]), the comparative fit index (CFI; value > .90 indicates reasonable fit; [43]), and the root-mean-square-error-of-approximation (RMSEA; value < .08 indicates reasonable fit; [44]). The analyses were conducted with R 3.0.2 and the Lavaan package [45].

#### Prevalence estimates

For the prevalence estimates, we tested a model with one latent factor *Z* predicting the prevalence of Crime, Loitering teens, Immigrants, Health care provision, Global warming, The recession, The government, The police, Corruption, Discrimination, Obesity, and Offensive behavior by strangers, on the personal versus collective level (note that the variables Personal safety, The economy, Money shortages, Unemployment, and Offensive behavior by friends were dropped from the analyses because preliminary analyses suggested item redundancies: personal safety was correlated .78 with crime; the economy, money shortages, and unemployment were correlated .89, .77 and .55 with the recession respectively; and offensive behavior by friends was correlated .78 with offensive behavior by strangers.) Two modification indices in preliminary analyses indicated above-average covariances. The models took this into account by including the covariance between the error terms of the variables Recession and The government, The police and Obesity in the model. Finally, the analyses of univariate skewness and kurtosis suggested possible violations of the assumption of multivariate normality. We therefore conducted analyses using maximum likelihood estimation with robust standard errors and a Satorra-Bentler scaled test statistic [35,46].

The fit indices indicated a good fit of the *Z*-model for collective-level estimates (CFI = .95; SRMR = .05; RMSEA = .06, 90% CI [.05; .08]), and poor to acceptable fit for personal-level estimates (CFI = .88; SRMR = .06; RMSEA = .05, 90% CI [.03; .06]). The amount of variance in the variables accounted for by *Z* was assessed by inspecting the *R* ^*2*^-values for all variables and calculating *R* ^*2*^-means (*R* ^*2*^-means were calculated by calculating the mean of Fisher Z transformed R-values, which were subsequently back-transformed and squared). As predicted, the mean *R* ^*2*^-value for the collective prevalence estimates was higher (*R* ^*2*^-mean = .45) than for personal prevalence estimates (*R* ^*2*^-mean = .29). Thus, results confirm that the one factor *Z*-model explained the largest amount of variance at the collective level.

#### Evaluative statements

Preliminary analyses of the statement-judgments indicated that positive and negative variables loaded onto separate factors, similar to Study 2. Accordingly we specified a bifactor model in CFA which modelled a positive and negative factor and (concurrently) the general factor *Z*. In such a bifactor model, all factors are independent (as opposed to a hierarchical model, is which the positive and negative factor would be correlated with the general factor) and this means that method variance can be partialled out. Moreover, in a bifactor approach the strength of the general factor (relative to the positive and negative factors) can be assessed (e.g., [47]).

First, model fit was briefly inspected. The fit indices suggested good fit of the bifactor model for *personal judgments* (CFI = .96; SRMR = .05; RMSEA = .07, 90% CI [.05; .09]) as well as for *collective judgments* (CFI = .94; SRMR = .05; RMSEA = .08, 90% CI [.06; .10]). Next, we inspected *R* ^*2*^-values for all items and calculated *R* ^*2*^-means accounted for by the models. Similar to results of Studies 1 and 2, somewhat less variance was accounted for by the model of personal judgments (*R* ^*2*^-mean = .45), than by the model of collective judgments (*R* ^*2*^ = .53). Within this overall variance explained, the positive and negative factors explained less variance (ranging from 19% to 21%) compared with the general factor: 59% for collective and 59% for personal, respectively. Putting things together, the results are consistent with those of Study 2: although there are differences between personally- and negatively worded items, there is also evidence for a general factor *Z* at the collective level in particular. But, it should be noted that compared with Study 2, the differences between the personal and collective level are smaller. The implications of this are considered in the discussion.

#### Predictive validity: the interpretation of news headlines

In a final set of analyses, we tested whether *Z* could predict the interpretation of negative news headlines. Similar to Study 2, we conducted a regression analysis with predictor *Z* represented by the average scores on the collective judgment dimensions. Hence, two scales were constructed by averaging scores on the prevalence estimates (as were used in CFA) on personal and collective level (P_30_: α = .78 and C_30_: α = .90). Furthermore, the two scales were constructed by averaging participants’ scores on the variables Violence, Egotism, Lack of decency, Injustice, Lack of respect and Corruption for personal-concrete and collective-abstract judgments (PC: α = .76 and CA: α = .81). We expected CA and C_30_ to be the best predictors in the model, both contributing to the interpretation of news headlines. The results are presented in Table 4.

**Table 4:** Regression Analysis for Predictors of Negative News Headlines (Study 3).

	B (SE)	β
Constant	3.85 (.22)***	
PC	-0.03 (.05)	-.04
P30	-0.02 (.01)	-.11
CA	0.27 (.06)	.31***
C30	0.03 (.01)	.26***
*R* ^2^	.19***	

*Note*. PC = personal-concrete judgments; P30 = personal prevalence estimates; CA = collective-abstract; C30 = collective prevalence estimates.

* *p* ≤ .05

** *p* < .01

*** *p* < .001.

Both collective-level judgment dimensions were significant predictors of people’s interpretation of negative news headlines, CA: β = .31, *t*(269) = 4.89, *p* < .001 and C_30_: β = .26, *t*(269) = 3.88, *p* < .001, while personal-level judgments were not. Thus, higher collective-level reports of problems in society again predicted higher likelihood of interpreting news headlines as truthful. The model significantly explained 19% of the variance in participants’ interpretation of news headlines (*F*(4, 269) = 15.28, *p* < .001). In line with the results of Study 2, this suggest that Z as represented by these collective constructs has predictive validity.

### Discussion

The results of Study 3 show that similar to what we found in Dutch society, our American participants were more negative and less positive about collective life than about their personal lives. In all, we again found evidence for our hypothesized conceptualization of Zeitgeist: for both types of collective-level judgments stronger evidence was found for a latent factor *Z* than for both types of personal-level judgments. Moreover, both collective-level measures predicted participants’ interpretation of news headlines.

One potential limitation of this study is the societal context at the time the data were collected: the US 2012 presidential elections. The salience of societal issues in discussion between politicians and in mass media during election time might have influenced people’s collective-level judgments of societal issues in our measures, potentially inflating the discrepancy between personal- and collective-level judgments that we found. However, given that discrepancy is very large and in line with the results of Study 1 and 2 we suspect that the interpretation of these findings, that participants were more negative about collective life than about their personal lives, remains valid.

Second, important to note is that this study showed that CFA procedures are extremely sensitive to item-overlap in the covariance matrix, that is to pairs or groups of items which correlated much higher with each other than with the other items. For example, high correlations between pairs of items in the prevalence-estimates measure (crime and personal safety, for example) prevented our initial CFAs from fitting acceptably and necessitated modifications to the CFA models (effectively reducing the influence of item redundancy). The sensitivity of these analyses to redundancy within the data demands careful item selection in future research.

Furthermore, while the results showed most evidence for a latent factor *Z* in collective-level statement judgments, the difference with personal-level judgments was relatively small. This difference was more pronounced between personal- and collective-level prevalence estimates: the *Z*-model had a good fit for collective estimates and accounted for a higher amount of the variance in the items, suggesting a greater influence of *Z* on people’s estimates of collective problems. Given the pattern of results that Study 3 provides, we recommend using the prevalence estimates method for measuring Zeitgeist in future research.

## General Discussion

In the present research we aimed to develop a new method of measuring Zeitgeist in order to better understand the current state of societal discontent in many Western countries. The results are consistent with the assumption that Zeitgeist is a collective global-level evaluation of the state (and future) of society that affects particular collective judgments concerning society. Across all three studies, we demonstrated that aggregated perceptions at the personal and collective level were very different (Study 1–3). Moreover, our theoretical model was supported by the present research: We consistently found evidence for a latent factor *Z*, underlying collective-level perceptions of the state of society (Study 1–3). In addition, the results showed that this Z predicted how new information about society is interpreted and attributed (Study 2 and 3).

The results confirm the apparent societal discontent that seems to characterize the Zeitgeist in both the Netherlands and the US: Our research showed a large significant difference between personal and collective perceptions of the same societal issues. On the more concrete prevalence estimates, Dutch and Americans alike believed that “the average person” in their country encountered societal problems many more times in the last 30 days than they did themselves. Moreover, the evaluative statement-judgments showed that while participants on average disagreed that societal issues pose a problem in their personal lives, they on average agreed that the same issues were a problem in society. The magnitude of these differences is unrealistically large, yet in line with what we anticipated to find about the Zeitgeist in those countries. Our findings suggest that to the respondents in our samples, both types of perceptions are “real” in the sense that these are the social realities they perceive in their personal life and in society in general. But it would be hard to ignore that collective-level perceptions both in the Netherlands and in the US are very different from realities that are grounded in concrete personal experience. While one might point out that personal-level perceptions are equally subjective and unlikely to correspond to “the truth” (and indeed we know they are distorted by various cognitive and motivational biases, see e.g. [5–7]), it is nevertheless important to note that personal-level perceptions are much more in line with official records of problems such as crime levels. These results are also consistent with the findings in the risk perception literature and cross-national and cross-cultural literature that point to the utility of measuring collective-level beliefs and values [18,19,21,23,24]. Even though such collective-level (or intersubjective) constructs do not necessarily bear any relation to their personal-level counterpart, they can uniquely predict variance in important outcomes. This stresses the importance of investigating and understanding collective-level perceptions.

Furthermore, the present research tested our hypothesized model of general factor *Z* underlying and predicting collective judgments about societal issues. The results indicated support for our approach: For collective-level judgments, we found one general latent factor *Z* (note that in the evaluative statement-measure in Study 2 and 3 a “method” factor reflected semantic differences between positively and negatively framed items). Noteworthy is that this latent *Z* factor predicts collective-level judgements across a broad range of topics, both currently important (e.g., the recession) and generally important in society (covering different moral foundations such as care, lack of respect, etc. [40,41]). In contrast, for personal-level judgments multiple factors were found, reflecting clusters of topics in various areas of life (e.g., safety, social bonds, economy, etc.). In addition, general factor *Z* explained the highest amount of variance in collective-level items in comparison to personal-level items, suggesting the strongest influence of *Z* on the various collective perceptions of society.

One unanticipated finding was the so-called “method factor” that appeared in the EFA for evaluative statements in Study 2 (and the preliminary analyses of Study 3). The valence of the items in these studies (positive vs. negative) created a second factor, because positive items were correlated slightly stronger with each other than with negative items and vice versa. Nevertheless, positive and negative items are also correlated strongly with each other and thus are anchored in a single dimension (see S1 Supplementary Materials), and most variance in the bifactor model in Study 3 was explained by general factor *Z*, as compared to either the positive or the negative factors. This suggests that the differences between positively- and negatively worded items are due mainly to the semantic differences between them. Nevertheless, it is also clear that positive and negative perceptions of societal issues are more independent than we initially assumed. A combination of focus group and survey based research shows that while people are mostly negative and easily identify problems in society, when pressed to think about things in society they can be proud of, they are able to come up with examples of topics that actually have considerable overlap with the identified problems (e.g. the way people live together in society, [1]). This indicates a possibly more complex relation between positive and negative perceptions of society, which could be an interesting topic for future research. But in view of the substantial and predicted correlation between the positive and negative judgments and the explained variance in the bifactor model in Study 3, our conclusion nevertheless is that the present results provide strong evidence for a general latent factor *Z*, underlying collective-level perceptions of society.

Importantly, Studies 2 and 3 demonstrated that *Z* can predict participants’ interpretation of new information about society. When collective perceptions were combined into a scale representing *Z*, this *Z* predicted participants’ interpretation of news headlines (Study 2 and 3) and the attribution of cause to society and humanity (Study 2). Not only do these results suggest that *Z* has predictive validity, they are also a first step towards investigating the possible influence of Zeitgeist on individual-level outcomes. At least, these results clearly indicate that collective perceptions are good predictors of individual responses to social information–better than personal perceptions in fact.

Summarizing these results, we suggest that this explorative research points to a new operationalization of societal discontents, conceived as Zeitgeist. Because the current climate of societal discontents was the starting point for this research, we have been focusing mostly on people’s negative views of what society is and on the problems they see in their country. However, theoretically we assume that Zeitgeist can be (overly) positive as well as (overly) negative, or somewhere in between. For example, until recently Australian citizens appear to have been quite optimistic about their country: In 2009, 49% of Australians were optimistic about the future (12% were pessimistic), and 48% reported to trust the federal government to “do the right thing for Australian people”[4]. Only recently this strongly changed: between 2010 and 2014, pessimism about the future has risen to 19% (optimism fell to 43%) and trust in government sharply declined to 26–31% [4]. While these statistics are hardly conclusive, they do point towards the possibility of (unrealistically high) optimism, in contrast to the unrealistically deep pessimism that we have seen some evidence of in the present research.

This assumption of relative independence between personal experiences and collective perceptions has important theoretical consequences. If personal experiences are (at least at certain historical junctures, and in relation to particular kinds of judgments) only tenuously relevant to collective perceptions, then how do these collective perceptions develop and change? Thus far, we can only make some educated guesses. First, people’s perceptions of society need to be *shared* for them to develop into collective evaluations, or ultimately Zeitgeist. Previous research has examined how collective perceptions such as stereotypes are influenced by consensualization in group interaction (e.g., [48–52]). Based on this research, we propose that the perceived consensus within society or some of its strata strongly affects the content and development of Zeitgeist. If perceived consensus is high, the Zeitgeist should be more pronounced, relatively stable and independent of personal perceptions. In the absence of perceived consensus about what “we” as society are, the Zeitgeist should be less pronounced, relatively unstable, and more open to the projection of personal perceptions.

Of course, it is often suggested that these shared perceptions are also strongly influenced by the media. But this mass communication effect is not as independent of consensualization effects as it may first appear: in many cases the influence of mass communication is filtered through personal communication [53–55]. Thus, people’s personal networks are likely to play a key role in shaping Zeitgeist, alongside mass media. The present data complicate this image somewhat further: respondents’ interpretation of news stories was strongly biased by their beliefs about society. Apparently, Zeitgeist also shapes our interpretations of news stories.

Finally, we suggest that in order to understand the factors shaping collective perceptions of the state of society, we need to devote some attention to people’s hopes and fears for their society’s (near) future. For example, a historical study of the Dutch Golden Age [56] suggests that periodic bouts of collective discontent during that time of great prosperity were fuelled by anticipated societal decline (i.e., a popular consensus, established and maintained in pamphlets, sermons and discourse, that sustained riches will eventually incur the wrath of God). In modern-day society, this implies that anticipation of societal change could predict the relative optimism or pessimism of Zeitgeist: concerns that current standards might not be sustainable or are under threat in the near future could lead to a more pessimistic Zeitgeist, that is the perception that society is in trouble today. In contrast, expectations of rising standards could lead to a more optimistic Zeitgeist. Both ideas, the perceived consensus and the perceived future state of society, open up interesting pathways for future research.

### Implications

Zooming in on the practical implications of this research, we believe that this work has relevance for public opinion research as well as for policy makers. Firstly, this research suggests that although the aggregation of people’s personal perceptions of societal issues provides information that is valuable and important in its own right, these statistics provide only part of the picture. Collective-level information is crucial to understanding interesting and complex societal phenomena. We provide some evidence for this in the domain of the discrepancy between personal well-being and collective discontent. This discrepancy currently can be observed in several countries around the world. But we see no reason why this measurement logic can not equally be applied to other phenomena (including those underlying collective euphoria prior to economic bubbles, the measurement of cultural difference, etc.). The reason for the importance of this approach is that while the content of the Zeitgeist might turn out to be not very “realistic”, the consequences of collective gloom about society are likely to be very real.

The present research showed that *collective* judgments of society (representing Zeitgeist) affect the interpretation of new information about society. If we extrapolate from this, we can be pretty sure that these perceptions shape and possibly distort “news” content, creating the specter of a social reality devoid of any resonance with real life. Ironically, this pessimistic Zeitgeist then would strongly affect politicians and policy makers to tackle societal problems which on closer inspection might exist mainly as figments of public imagination. Anyone (politician, policy maker, scientist or journalist) who advocates that policy should be based on crime statistics and hard evidence, is likely to encounter the charge of being out of touch with the “reality” of crime and safety perceptions. Our social psychological approach could help to understand and disentangle such societal pressures. If for example people demand more crime fighting because they feel unsafe, while actual crime rates have been consistently dropping over the years (as is the case in the Netherlands and was the case in the USA during the 1990s), we suggest the “problem” to exist mainly as a collective perception of society being unsafe; to be effective, solutions should then address these collective perceptions, not (only) personal experiences. And in debates surrounding these issues, it may help to steer discussions away from the general collective level, and towards the concrete personal level: after all, the same people who believe that collective problems abound are likely to be quite content in their personal life, in their neighborhood, at work, etc. It is at this concrete local level that perceptions and policy choices may best coincide.

To conclude, this research is an important first step towards the development of a social psychological approach of Zeitgeist, as a collective global-level affective evaluation of the state of society. We found evidence in our studies for a large discrepancy between personal and collective perceptions of the same societal issues, confirming gloominess about the state of society that appears to exist in the Netherlands and the US. Furthermore, we have developed two new methods of measuring Zeitgeist as general factor *Z*, which underlies and colors collective judgments of specific societal issues. Finally, we demonstrated that *Z* can predict people’s interpretation of new information about society. While the present work cannot answer all important questions that it raises, we believe it to be of great value to both researchers and policy makers as it helps to disentangle this puzzling societal discontent and keeping two levels of discontent (personal and collective) separate. The present work may be considered a first contribution to a new research agenda, aiming to understand the importance of Zeitgeist to society.

## Supporting Information

S1 Appendix (DOCX)

S1 Supplementary Materials (DOCX)
