# Supplementary material for: Understanding Collective Discontents: A Psychological Approach to Measuring Zeitgeist
Source: PLoS One. 2015 Jun 26;10(6):e0130100. doi: 10.1371/journal.pone.0130100 (PMC4482588; doi:10.1371/journal.pone.0130100)
Supplement: S1 Appendix — (DOCX) [file pone.0130100.s001.docx]

**Appendix**

**Prevalence estimates – English (Study 3)**

**Personal-level instructions:**

Please think about your daily life in the last 30 days.

Think about the conversations you had, the things you did, the people and situations you encountered during the last 30 days. What problems did you encounter in your personal life during these last 30 days?

How many of the last 30 days did you encounter problems with…

Please, fill in a number between 0 and 30 (days):

**Collective-level instructions:**

Next, please think about the life of the average American.

How does the life of an American person look during 30 days? Think about the conversations that Americans have, the things they do, the people and situations they encounter. What kind of problems does an average American person encounter during the last 30 days?

How many out of the last 30 days did the average American encounter problems with…

Please, fill in a number between 0 and 30 (days):

**Items:**

Crime

Personal safety

Loitering teens

Immigrants

Health care provision

Global warming

The economy

The recession

Money shortages or budget cuts

Unemployment

The government

The police

Corruption or fraud

Discrimination

Obesity or being overweight

Alcohol or drugs abuse in your immediate environment

Indecent or antisocial behavior by strangers

Indecent or antisocial behavior by friends/acquaintances

**Evaluative statements – English (Study 3)**

**Personal-level items**

*Theme*

Personal-concrete item.

Personal-abstract item.

Instructions:

**Personal day to day experiences.**

**The following questions concern you and your personal life. Think about the conversations you have, the things you do, the people and situations you encounter in your daily life.**

**Please indicate to what extent you agree with each of the following statements, by selecting a number from 1 (completely disagree) to 7 (completely agree), with 4 indicating that you neither agree nor disagree.**

*Violence*

I regularly encounter violent behavior in the streets.

Violence is a problem in my life.

*Egotism*

In my experience, people act mainly out of self-interest.

Egotism is a problem in my personal life.

*Honesty*

In general, I am treated honestly.

In my personal life, people are generally honest.

*Trust*

I trust the people I know.

In my personal life, people are generally trustworthy.

*Inequality*

Inequality is a problem in my personal life.

I personally know people who get more than they deserve.

*Care*

In my personal life it is the case that when something happens to me, I will be looked after.

In my personal life, people look after each other.

*Injustice*

I am often treated unjustly by others.

Injustice is a problem in my personal life.

*Lack of respect*

In my personal life, people treat me without respect.

Lack of respect is a problem in my personal life.

*Social cohesion*

In my immediate environment, I see strong social cohesion.

In my immediate environment, many people have strong bonds with each other.

*Corruption*

Many people I know act corruptly.

In my personal life, I see a lot of corruption.

*Loyalty*

In my personal life, people I know are loyal to each other.

In my personal life, people are loyal.

*Lack of decency*

I regularly encounter indecent behavior of others.

I experience the indecency of others as a personal problem.

**Collective-level items**

*Theme*

Personal-concrete item.

Personal-abstract item.

Instructions:

**Experiences in society.**

**The following questions concern present-day society. Think about the issues that other Americans talk about, the things other Americans do and the people and situations that other Americans encounter in American society.**

**Please indicate to what extent you agree with each of the following statements, by selecting a number from 1 (completely disagree) to 7 (completely agree), with 4 indicating that you neither agree nor disagree.**

*Violence*

Americans regularly encounter violent behavior in the streets.

Violence is a national problem in the U.S.

*Egotism*

Egotism is a national problem in the U.S.

Many Americans act mostly out of self-interest.

*Honesty*

In general, Americans treat each other honestly.

Americans are honest.

*Trust*

Americans generally trust each other.

Americans are generally trustworthy.

*Inequality*

In American society, some people get more than they deserve.

Inequality is a national problem in American society.

*Care*

In the U.S., it is the case that when something happens to you, you will be looked after.

In the U.S., people are well looked after.

*Injustice*

Americans often treat each other unjustly.

Injustice is a national problem in the U.S.

*Lack of respect*

In American society, people treat each other without respect.

American society is characterized by a lack of respect.

*Social cohesion*

In American society, many people have strong bonds with each other.

In American society, there is strong social cohesion.

*Corruption*

Many Americans act corruptly.

In American society, there is a lot of corruption.

*Loyalty*

Americans are generally loyal to each other.

In American society, people are loyal.

*Lack of decency*

Americans regularly encounter indecent behavior of others.

Indecency is a national problem in the U.S.

**Prevalence estimates – Dutch (Study 1)**

**[Personal-level instructions:]**

De volgende vragen gaan over jouw persoonlijke leven gedurende de afgelopen 30 dagen. Denk aan de gesprekken die je voerde, de dingen die je deed, de mensen en situaties die je tegenkwam.

Hoeveel van de afgelopen 30 dagen heb jij last gehad van...

**[Collective-level instructions:]**

De volgende vragen gaan over de gemiddelde Nederlander. Hoe ziet het leven van een Nederlander er gedurende 30 dagen uit? Denk aan de gesprekken die Nederlanders voeren, de dingen die ze doen, de mensen en situaties die ze tegenkomen.

Hoeveel van de afgelopen 30 dagen heeft een Nederlander last van...

**[Items:]**

|  | Vul een cijfer tussen 0 en 30 (dagen) in: |
| --- | --- |
| Criminaliteit |  |
| Alcoholmisbruik in de directe omgeving |  |
| Immigranten |  |
| Hangjongeren |  |
| De recessie |  |
| Zelfverrijking of fraude |  |
| De overheid |  |
| De politie |  |
| Allochtonen |  |
| Bezuinigingen of geldtekort |  |
| Onfatsoenlijk of asociaal gedrag van bekenden |  |
| Onfatsoenlijk of asociaal gedrag van onbekenden |  |

**Evaluative statements – Dutch (Study 2)**

**Personal-level items - Dutch**

*Theme*

Personal-concrete item.

Personal-abstract item.

Instructions:

**De volgende vragen gaan over u en uw persoonlijke leven.**

**Denk bijvoorbeeld aan de gesprekken die u voert, de dingen die u doet, de mensen en situaties die u tegenkomt in uw dagelijks leven.**

**Geef aan in hoeverre u het eens bent met de volgende stellingen door een cijfer te kiezen op een schaal van 1 (*helemaal oneens*) tot 7 (*helemaal eens*), waarbij 4 staat voor niet eens/niet oneens. Wanneer u een antwoord niet weet of een vraag liever niet wilt beantwoorden, kunt u de vraag open laten.**

*Gewelddadigheid* [Violence]

Ik heb last van geweld op straat.

Gewelddadigheid is een probleem in mijn leven.

*Zorgen voor/bekommeren om elkaar* [Care]

In mijn persoonlijke leven is het zo dat als je iets overkomt, er voor je wordt gezorgd.

In mijn persoonlijke leven wordt goed voor mensen gezorgd.

*Eerlijkheid* [Honesty]

Ik word over het algemeen eerlijk behandeld.

In mijn persoonlijke leven zijn mensen eerlijk.

*Corruptie* [Corruption]

Ik ken mensen die corrupt handelen.

Ik zie in mijn persoonlijke leven veel corruptie.

*Onrechtvaardigheid (anderen/algemeen)* [Injustice by others]

Ik word vaak onrechtvaardig behandeld door andere mensen.

Onrechtvaardigheid is een probleem in mijn persoonlijke leven.

*Onrechtvaardigheid (instanties)* [Injustice by governmental agencies]

Overheidsinstanties behandelen mij vaak op een onrechtvaardige manier.

Onrechtvaardigheid door overheidsinstanties is een probleem in mijn persoonlijke leven.

*Ongelijkheid* [Inequality]

Ik ken persoonlijk mensen die op een oneerlijke manier meer krijgen dan anderen.

Ongelijkheid is een probleem in mijn persoonlijke leven.

*Vertrouwen* [Trust]

Mensen in mijn persoonlijke leven gedragen zich betrouwbaar jegens elkaar.

In mijn persoonlijke leven zijn mensen betrouwbaar.

*Sociale cohesie* [Social cohesion]

In mijn omgeving hebben veel mensen een sterke band met elkaar.

In mijn omgeving zie ik een sterke sociale verbondenheid.

*Egoïsme* [Egotism]

In mijn persoonlijke leven ervaar ik dat mensen vooral uit eigenbelang handelen.

Egoïsme is een probleem in mijn persoonlijke leven.

*Immigratie* [Immigration]

In mijn directe omgeving heb ik last van allochtonen.

Immigratie is een probleem in mijn directe omgeving.

*Loyaliteit* [Loyalty]

In mijn omgeving zijn mensen trouw aan elkaar.

In mijn omgeving zijn mensen loyaal.

*Gebrek aan respect* [Lack of respect]

In mijn directe omgeving behandelen mensen elkaar zonder respect.

In mijn directe omgeving zie ik een gebrek aan respect.

*Gebrek aan fatsoen* [Lack of decency]

In mijn persoonlijke leven heb ik last van onfatsoenlijk gedrag van anderen.

In mijn persoonlijke leven is gebrek aan fatsoen een probleem.

**Collective-level items - Dutch**

*Theme*

Collective-concrete item.

Collective-abstract item.

Instructions:

**De volgende vragen gaan over de Nederlandse samenleving.**

**Denk bijvoorbeeld aan de gespreksonderwerpen waar Nederlanders veel over praten, de dingen die Nederlanders doen en de mensen en situaties die Nederlanders tegenkomen in de Nederlandse samenleving.**

**Geef aan in hoeverre u het eens bent met de volgende stellingen door een cijfer te kiezen op een schaal van 1 (*helemaal oneens*) tot 7 (*helemaal eens*), waarbij 4 staat voor niet eens/niet oneens. Wanneer u een antwoord niet weet of een vraag liever niet wilt beantwoorden, kunt u de vraag open laten.**

*Gewelddadigheid* [Violence]

Nederlanders hebben last van geweld op straat.

Gewelddadigheid is een probleem in Nederland.

*Zorgen voor/bekommeren om elkaar* [Care]

In de samenleving is het zo dat als je iets overkomt, dan wordt er voor je gezorgd.

In de samenleving wordt goed voor mensen gezorgd.

*Eerlijkheid* [Honesty]

Nederlanders worden over het algemeen eerlijk behandeld.

In Nederland zijn mensen eerlijk.

*Corruptie* [Corruption]

In de Nederlandse samenleving zijn mensen die corrupt handelen.

In de Nederlandse samenleving is veel corruptie.

*Onrechtvaardigheid (anderen/algemeen)* [Injustice by others]

Nederlanders worden vaak onrechtvaardig behandeld door andere mensen.

Onrechtvaardigheid is een probleem in Nederland.

*Onrechtvaardigheid (instanties)* [Injustice by governmental agencies]

Overheidsinstanties behandelen Nederlanders vaak op een onrechtvaardige manier.

Onrechtvaardigheid door overheidsinstanties is een probleem in Nederland.

*Ongelijkheid* [Inequality]

In de samenleving krijgen sommige mensen meer dan gelijke anderen.

Ongelijkheid is een probleem in de samenleving.

*Vertrouwen* [Trust]

Nederlanders gedragen zich betrouwbaar jegens elkaar.

In de Nederlandse samenleving zijn mensen betrouwbaar.

*Sociale cohesie* [Social cohesion]

In de Nederlandse samenleving hebben veel mensen een sterke band met elkaar.

In de Nederlandse samenleving is een sterke sociale verbondenheid.

*Egoïsme* [Egotism]

In de samenleving is de ervaring dat mensen vooral uit eigenbelang handelen.

Egoïsme is een probleem in de samenleving.

*Immigratie* [Immigration]

Nederlanders hebben last van allochtonen.

Immigratie is een probleem in Nederland.

*Loyaliteit* [Loyalty]

In de Nederlandse samenleving zijn mensen trouw aan elkaar.

In de Nederlandse samenleving zijn mensen loyaal.

*Gebrek aan respect* [Lack of respect]

In de samenleving behandelen mensen elkaar zonder respect.

De samenleving wordt gekenmerkt door een gebrek aan respect.

*Gebrek aan fatsoen* [Lack of decency]

Nederlanders hebben last van onfatsoenlijk gedrag van anderen.

Gebrek aan fatsoen is een probleem in Nederland.
