# Supplementary material for: Understanding Collective Discontents: A Psychological Approach to Measuring Zeitgeist
Source: PLoS One. 2015 Jun 26;10(6):e0130100. doi: 10.1371/journal.pone.0130100 (PMC4482588; doi:10.1371/journal.pone.0130100)
Supplement: S1 Supplementary Materials — (DOCX) [file pone.0130100.s002.docx]

**Supplementary Materials**

**Abstract versus Concrete Statements (Study 2)**

In the evaluative statement measure in Study 2, we included not only personal-concrete and collective-abstract statements of the same societal issues, but for explorative reasons also personal-abstract and collective-concrete statements. In this way we aimed to disentangle the influence of the personal-collective dimension from the concrete-abstract dimension. These results are discussed below.

**Differences in means**

The full 2 (personal vs. collective) by 2 (concrete vs. abstract) repeated measures ANOVA’s were conducted separately on positively- and negatively-framed topics. For negative topics, the repeated measures ANOVA showed two significant main effects (of which the personal-collective main effect was the strongest, *F*(1,247) = 644.20, *p* < .001, η_p_^2^ = .72), qualified by a significant interaction, (*F*(1,247) = 20.78, *p* < .001, η_p_^2^ = .08). While there was no difference between collective-concrete (*M* = 4.60, 95% CI [4.50, 4.70]) and collective-abstract judgments (*M* = 4.60, 95% CI [4.49, 4.72]), participants disagreed somewhat more with the personal-abstract topics (*M* = 3.12, 95% CI [2.99, 3.25]), than with the personal-concrete topics (*M* = 3.31, 95% CI [3.17, 3.44]).

For positive topics, a strongly significant main effect of the personal-collective dimension was found, *F*(1,247) = 195.52, *p* < .001, η_p_^2^ = .44. Participants on average indicated stronger agreement that positive topics affected their personal life, *M* = 4.81 (95% CI [4.68, 4.94]), than they did for collective life, *M* = 4.12, (95% CI [4.00, 4.24]). The main effect of the concrete-abstract dimension and the interaction effect were non-significant (both *F’s* < 1).

**Exploratory Factor Analyses (EFA)**

The EFAs showed that the level of abstractness of the statements was important in determining factor structure. The factor structure of the *collective-concrete judgments* was more similar to that of the personal-concrete judgments, than that of the collective-abstract judgments: three factors, with eigenvalues of 4.79, 2.11, and 1.16 and 57.55% explained variance. Positive topics were one separate factor. Negative topics formed two factors, one of which appeared to consist of more concrete problems (e.g., immigration, violence) and one of more abstract problems (e.g., inequality). With factor loadings ranging from .40 to .85 (only two factor loadings < .45), this factor structure seems to fit the data well.

The factor structure of the *personal-abstract judgments* was more similar to the collective-abstract judgments: two factors were extracted with eigenvalues of 5.38 and 1.46, resembling the collective-abstract factor structure. One factor consisted of all positive items plus “Lack of respect”; the other of all remaining negative items. The correlation between these factors was .66. The total explained variance was 48.90%, and the solution was slightly less well-defined: factor loadings ranged from .37 to .81 and communality coefficients were somewhat lower on average (e.g., four coefficients < .40). This two-factor solution therefore seems a reasonable fit to the data.

**Assessing variance explained by Z**

The influence of *Z* on the judgments of societal issues was investigated by calculating the amount of variance in the items that was accounted for by *Z* (i.e., in this model *Z-negative* and *Z-positive*). Interestingly, for both hybrid dimensions (collective-concrete and personal-abstract judgments), on average less variance in the items was explained by Z compared to both other dimensions: for negative items 35% for collective-concrete and 34% for personal-abstract statements; and for positive items 51% and 51%, respectively.

**Discussion**

These results showed that the personal-abstract and collective-concrete judgment dimensions shared some characteristics of personal-concrete judgments and other characteristics of the collective-abstract judgments. For the pattern of the mean differences the most important aspect is whether the judgments were made at personal versus collective level. On the other hand, it appears that the abstractness of the concepts that are judged determines the degree to which they cohere. These findings extend insights presented in the paper in one respect: they confirm that personal-concrete and collective-abstract judgments are indeed two distinct categories of judgment, even if they appear to refer to the same topics or issues.

**Relations between personal and collective judgments (Studies 1-3)**

Across all studies, we inspected correlations between the various personal- and collective-level judgments to explore the relation between them. Table D presents these correlations. These results show that the correlation between personal- and collective-level judgments (within the same measures) is consistently moderate to strong. Positive statement-judgments are correlated stronger with each other than negative statement-judgments. These analyses confirm that personal-level and collective-level judgments share a limited amount of overlap.

**Table A. Correlations between Personal and Collective judgments and Additional Measures, Study 1 (N = 166).**

|  | 1 | 2 | 3 | 4 | 5 | 6 | 7 | 8 |
| --- | --- | --- | --- | --- | --- | --- | --- | --- |
| 1. Collective | -- |  |  |  |  |  |  |  |
| 2. Personal Factor 1 | .42*** | -- |  |  |  |  |  |  |
| 3. Personal Factor 2 | .37*** | .26** | -- |  |  |  |  |  |
| 4. Personal Factor 3 | .24** | .21** | .08 | -- |  |  |  |  |
| 5. Personal Factor 4 | .48*** | .39*** | .24** | .26** | -- |  |  |  |
| 6. BJW - God | -.03 | .06 | -.03 | -.13 | .00 | -- |  |  |
| 7. BJW - Nature | .04 | -.03 | -.07 | -.08 | -.01 | .28*** | -- |  |
| 8. Life satisfaction | -.09 | .02 | -.16* | .15 | .09 | .01 | -.01 | -- |
| 9. Personal optimism | -.13 | -.13 | -.15 | .05 | -.10 | -.14 | -.12 | .30*** |

*Note.* Collective = mean of collective-level prevalence estimates (items as in EFA); Personal Factor 1-4 = mean of personal-level prevalence estimates EFA Factor 1 to 4; BJT – God = mean score of Belief in a Just Treatment scale, God as source of justice (7 items, adapted from [1]); BJT – Nature = mean score on Belief in a Just Treatment scale, Nature as source of justice (7 items, adapted from [1]); Life satisfaction = mean score on satisfaction with life scale (5 items, adapted from [2]); Personal optimism = mean score on optimism (6 items, adapted from [3]).

* *p* ≤ .05; ** *p* < .01; *** *p* < .001.

**Table B. Correlations between Personal and Collective judgments and Additional Measures, Study 2 (N = 248).**

|  | 1 | 2 | 3 | 4 | 5 | 6 | 7 | 8 | 9 | 10 | 11 |
| --- | --- | --- | --- | --- | --- | --- | --- | --- | --- | --- | --- |
| 1. Collective Factor 1 | -- |  |  |  |  |  |  |  |  |  |  |
| 2. Collective Factor 2 | -.47*** | -- |  |  |  |  |  |  |  |  |  |
| 3. Personal Factor 1 | .58*** | -.26*** | -- |  |  |  |  |  |  |  |  |
| 4. Personal Factor 2 | -.41*** | .49*** | -.37*** | -- |  |  |  |  |  |  |  |
| 5. Personal Factor 3 | -.15* | .34*** | -.25*** | .42*** | -- |  |  |  |  |  |  |
| 6. Personal optimism | .43*** | -.33*** | .42*** | -.48*** | -.31*** | -- |  |  |  |  |  |
| 7. Identification | .49*** | -.23*** | .47*** | -.19** | -.10 | .24*** | -- |  |  |  |  |
| 8. Neighborhood safety | -.22*** | .21** | -.27*** | .31*** | .55*** | -.35*** | -.19** | -- |  |  |  |
| 9. General social trust | .53*** | -.35*** | .41*** | -.39*** | -.26*** | .37*** | .26*** | -.23*** | -- |  |  |
| 10. Trust in other groups | .42*** | -.29*** | .32*** | -.36*** | -.41*** | .33*** | .21** | -.33*** | .58*** | -- |  |
| 11. Political trust | .62*** | -.52*** | .39*** | -.33*** | -.15* | .36*** | .42*** | -.26*** | .48*** | .47*** | -- |
| 12. State of the country | .48*** | -.40*** | .22** | -.22*** | -.04 | .25*** | .30*** | -.13* | .26*** | .30*** | .64*** |

*Note.* Collective Factor 1 = mean of collective-abstract evaluative statements in EFA Factor 1; Collective Factor 2 = mean of collective-abstract evaluative statements in EFA Factor 2; Personal Factor 1-3 = mean of personal-concrete evaluative statements in EFA Factor 1 to 3; Personal optimism = mean of optimism scale (6 items; adapted from [3]); Identification = mean identification with the Netherlands, 4 item-scale (FISI; [4]); Neighbourhood safety = mean of 4 item-scale, adapted from [5]; General social trust = mean of 3 item-scale adapted from [5]; Social trust in other groups = mean of 4 item-scale adapted from [6]; Political trust = mean of 4 item-scale adapted from [5]; State of the country = mean of 2 items: “In general, would you expect life for most people in the Netherlands to become better or worse?” and “To what extent is life for most people in the Netherlands better or worse than in the year 2000?”.

* *p* ≤ .05; ** *p* < .01; *** *p* < .001.

**Table C. Correlations between Personal and Collective judgments and Additional Measures, Study 3 (N = 275).**

|  | 1 | 2 | 3 | 4 | 5 | 6 | 7 | 8 | 9 | 10 |
| --- | --- | --- | --- | --- | --- | --- | --- | --- | --- | --- |
| 1. C30 | -- |  |  |  |  |  |  |  |  |  |
| 2. P30 | .54*** | -- |  |  |  |  |  |  |  |  |
| 3. CA *neg.* | .41*** | .28*** | -- |  |  |  |  |  |  |  |
| 4. CA *pos.* | -.27*** | -.26*** | -.52*** | -- |  |  |  |  |  |  |
| 5. PC *neg.* | .27*** | .35*** | .39*** | -.43*** | -- |  |  |  |  |  |
| 6. PC *pos.* | -.22*** | -.25*** | -.24*** | .53*** | -.54*** | -- |  |  |  |  |
| 7. Personal optimism | -.22*** | -.21*** | -.30*** | .46*** | -.37*** | .49*** | -- |  |  |  |
| 8. Neighborhood safety | .15* | .25*** | .34*** | -.34*** | .42*** | -.23*** | -.24*** | -- |  |  |
| 9. General social trust | -.23*** | -.22*** | -.35*** | .60*** | -.40*** | .52*** | .49*** | -.31*** | -- |  |
| 10. Trust in other groups | -.19** | -.18** | -.16** | .45*** | -.31*** | .44*** | .35*** | -.16** | .59*** | -- |
| 11. Political trust | -.19** | -.17** | -.20** | .39*** | -.11 | .29*** | .22*** | -.03 | .25*** | .26*** |

*Note.* C30 = mean of collective-level prevalence estimates; P30 = mean of personal-level prevalence estimates; CA *neg*. = mean of negative collective-abstract evaluative statements; CA *pos*. = mean of positive collective-abstract evaluative statements; PC *neg*. = mean of negative personal-concrete evaluative statements; PC *pos*. = mean of positive personal-concrete evaluative statements; Personal optimism = mean of optimism scale (6 items; [3]); Identification = mean identification with the Netherlands, 4 item-scale (FISI; [4]); Neighbourhood safety = mean of 4 item-scale, [5]; General social trust = mean of 3 item-scale [5]; Social trust in other groups = mean of 4 item-scale [6]; Political trust = mean of 4 item-scale [5].

* *p* ≤ .05; ** *p* < .01; *** *p* < .001.

**Table D.** **Correlations between Dimensions (Study 1-3)**

|  | Study 1 | | Study 2 | | | | Study 3 | | | | | |
| --- | --- | --- | --- | --- | --- | --- | --- | --- | --- | --- | --- | --- |
|  | P30 | C30 | PC *neg*. | CA *neg*. | PC *pos*. | CA *pos*. | P30 | C30 | PC *neg*. | CA *neg*. | PC *pos*. | CA *pos*. |
| *Study 1* |  |  |  |  |  |  |  |  |  |  |  |  |
| P30 | – |  |  |  |  |  |  |  |  |  |  |  |
| C30 | .58 | – |  |  |  |  |  |  |  |  |  |  |
| *Study 2* |  |  |  |  |  |  |  |  |  |  |  |  |
| PC *neg.* |  |  | – |  |  |  |  |  |  |  |  |  |
| CA *neg.* |  |  | .53 | – |  |  |  |  |  |  |  |  |
| PC *pos.* |  |  | -.56 | -.33 | – |  |  |  |  |  |  |  |
| CA *pos.* |  |  | -.40 | -.47 | .63 | – |  |  |  |  |  |  |
| *Study 3* |  |  |  |  |  |  |  |  |  |  |  |  |
| P30 |  |  |  |  |  |  | – |  |  |  |  |  |
| C30 |  |  |  |  |  |  | .54 | – |  |  |  |  |
| PC *neg.* |  |  |  |  |  |  | .35 | .27 | – |  |  |  |
| CA *neg.* |  |  |  |  |  |  | .28 | .41 | .39 | – |  |  |
| PC *pos.* |  |  |  |  |  |  | -.25 | -.22 | -.54 | -.24 | – |  |
| CA *pos.* |  |  |  |  |  |  | -.26 | -.27 | -.43 | -.51 | .53 | – |

*Note.* All correlations are significant *p* < .01. P30 = personal prevalence estimates; C30 = collective prevalence estimates; PC = personal-concrete statement judgments; CA = collective-abstract statement judgments; *neg.* = negative; *pos.* = positive.

**References**

1. Stroebe K, Postmes T, Täuber S, Stegeman A, John M-S. Belief in a Just What? Demystifying Just World Beliefs by Distinguishing Sources of Justice. PLoS One. Public Library of Science; 2015;10: e0120145. Available: http://dx.doi.org/10.1371%2Fjournal.pone.0120145

2. Diener E, Emmons RA, Larsen RJ, Griffin S. The Satisfaction With Life Scale. J Pers Assess. US: Lawrence Erlbaum; 1985;49: 71–75. doi:10.1207/s15327752jpa4901_13

3. Scheier MF, Carver CS, Bridges MW. Distinguishing optimism from neuroticism (and trait anxiety, self-mastery, and self-esteem): A reevaluation of the Life Orientation Test. J Pers Soc Psychol. US: American Psychological Association; 1994;67: 1063–1078. doi:10.1037/0022-3514.67.6.1063

4. Postmes T, Haslam SA, Jans L. A single‐item measure of social identification: Reliability, validity, and utility. Br J Soc Psychol. 2013;52: 597–617. doi:10.1111/bjso.12006

5. European Social Survey. ESS-6 2012 Survey [Internet]. Bergen, Norway; 2012. Available: http://www.europeansocialsurvey.org/data/download.html?r=6

6. World Value Survey. World Value Survey Wave 6 2010-2014 - Survey [Internet]. 2012. Available: http://www.worldvaluessurvey.org/WVSDocumentationWV6.jsp
